# Supplementary material for: Retbindin: A riboflavin Binding Protein, Is Critical for Photoreceptor Homeostasis and Survival in Models of Retinal Degeneration
Source: Int J Mol Sci. 2020 Oct 29;21(21):8083. doi: 10.3390/ijms21218083 (PMC7662319; doi:10.3390/ijms21218083)
Supplement: Supplementary file 1 [file ijms-21-08083-s001.pdf]

# Supplementary Information

## Retbindin, a riboflavin binding protein, is critical for photoreceptor homeostasis and survival in multiple models of retinal degeneration

Ayse M. Genc[1], Mustafa S. Makia[1], Tirthankar Sinha[1], Shannon M. Conley[2,3], Muayyad R. Al-Ubaidi[*1,4,5], and Muna I. Naash[*1,4,5]

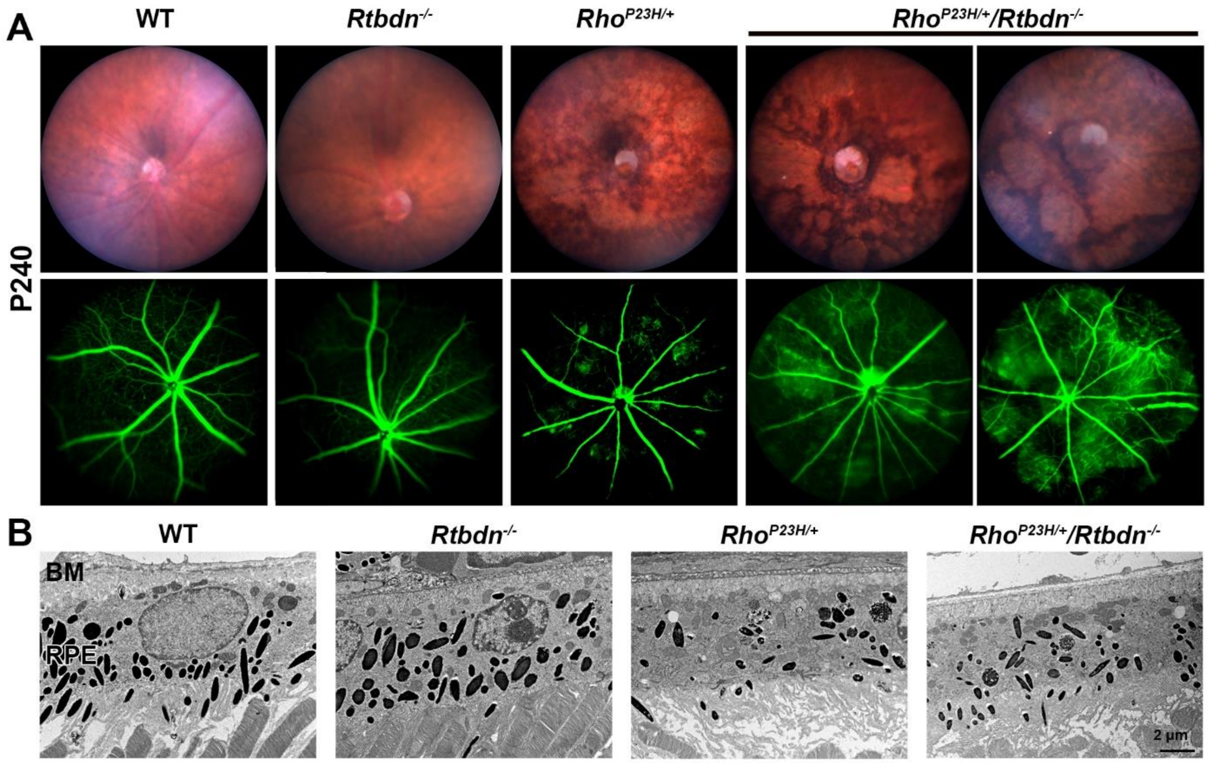


**Figure S1**. **Non-photoreceptor effects of eliminating *Rtbdn* in aged *Rho*$^{P23H/+}$ eyes**. (A) Shown are bright field fundus (top) and fluorescein angiograms (bottom) from mice of indicated genotypes at P240. Two different *Rho*$^{P23H/+}$/*Rtbdn*$^{-/-}$ are displayed. (B) TEM from P30 eyes of the indicated genotypes showing the RPE and Bruch's membrane. BM, Bruch's membrane; RPE, retinal pigment epithelium. Scale bar: 2 µm, magnification 5,000x.

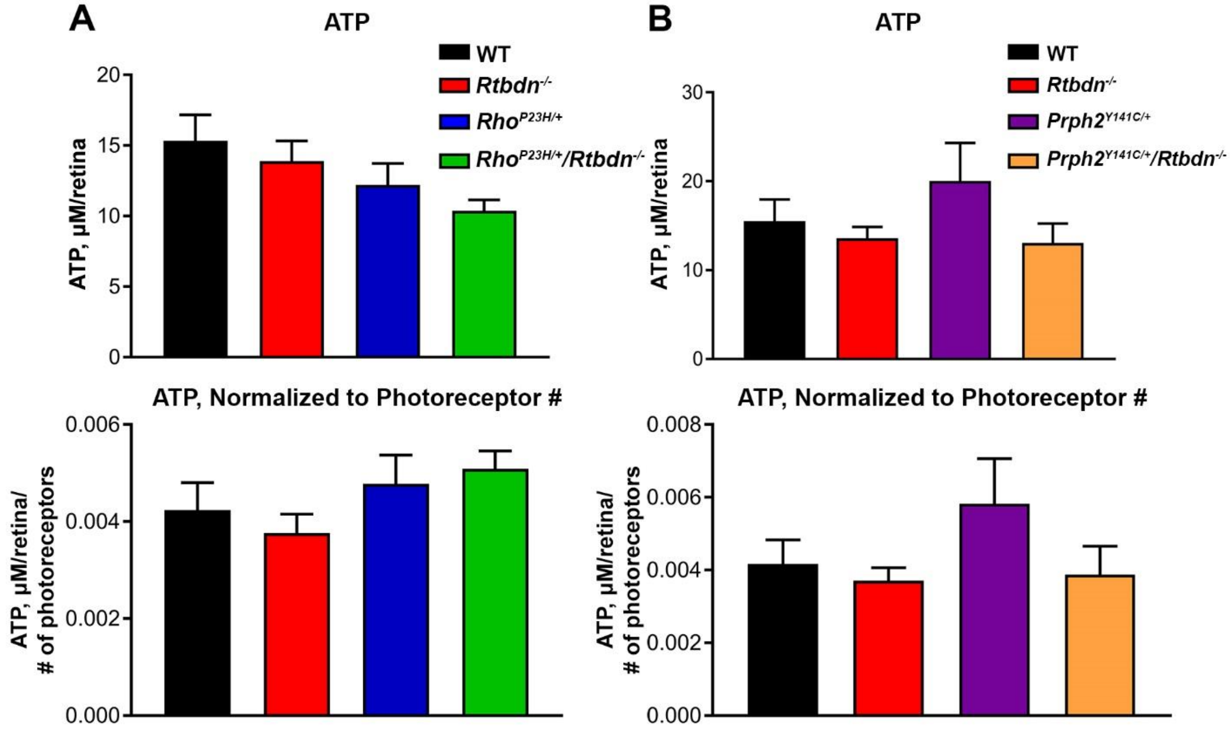


**Figure S2. ATP levels are unaffected in degenerating retinas**. (A-B) ATP levels were measured at P30 in retinas of the indicated genotypes (N=6-15 retinas/group). Data were plotted as +SEM. Bottom panel in each represents ATP levels normalized to the number of remaining photoreceptors.
